# A Bio‐Adaptive Janus‐Adhesive Dressing with Dynamic Lubrication Overlayer for Prevention of Postoperative Infection and Adhesion

**DOI:** 10.1002/advs.202500138

**Published:** 2025-03-20

**Authors:** Yuan Gao, Junchang Guo, Shuangyang Li, Liansong Ye, Binyang Lu, Jiaxin Liu, Jing Luo, Yijia Zhu, Liuxiang Chen, Tingfa Peng, Jinlong Yang, Dehui Wang, Chaoming Xie, Xu Deng, Bing Hu

**Affiliations:** ^1^ Department of Gastroenterology and Hepatology Digestive Endoscopy Medical Engineering Research Laboratory West China Hospital Med‐X Center for Materials Sichuan University Chengdu 610064 P. R. China; ^2^ Institute of Fundamental and Frontier Sciences University of Electronic Science and Technology of China Chengdu 610054 P. R. China; ^3^ Key Lab of Advanced Technologies of Materials Ministry of Education School of Materials Science and Engineering Southwest Jiaotong University Chengdu 610031 P. R. China

**Keywords:** antifouling, Janus‐adhesive gel, liquid‐infused surface, postoperative adhesion

## Abstract

Wound postoperative infection and adhesion are prevalent clinical conditions resulting from surgical trauma. However, integrating intraoperative repair and postoperative management into a dressing suitable for wounds with unpredictable surface shapes and surroundings remains a formidable challenge. Here, we attempt to introduce a dynamic antifouling surface as wound protective covering and report an in situ formation of slippery‐adhesive Janus gel (SAJG) by assembling hydrogel (N‐hydrosuccinimide ester‐activated powders) and elastomer (Silicon oil‐infused polydimethylsiloxane). First powders can rapidly absorb interfacial water to gel and bond to tissue based on network entanglement, forming a tough adhesive hydrogel. Then precured organosilicon is applied to hydrogel and bonded together, forming a slippery elastomer. Due to the molecular polarity difference between hydrogel and elastomer, SAJG exhibits anisotropic surface behavior as evidenced by liquid repellency (hydrophilic vs. hydrophobic), and adhesion performance (bioadhesion vs. antiadhesion). Further, in vivo models are constructed and results demonstrated that the SAJG can effectively prevent bacterial infection to promote wound healing and avoid postoperative adhesion. Predictably, the morphologically adaptive SAJG with slippery and adhesive properties will have tremendous potential in addressing complex wound infections and postoperative complications.

## Graphical abstract

Slippery‐adhesive Janus gel dressing exhibits both stable tissue adhesive and outstanding efficiency in repelling physiological fluids. As a physical barrier, a liquid‐infused surface with a dynamic lubrication overlayer possesses excellent antibiofouling properties, making it highly effective in preventing bacterial infections and postoperative tissue adhesions.

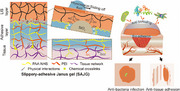

## 1 Introduction

Infection and adhesion as common postoperative complications are the result of microbial erosion and abnormal fibrous tissue formation during tissue injury and subsequent healing.^[^ ^1^ ^]^ Clinically, the most common non‐surgical approach to prevent postoperative infection and adhesion is drug treatment and biomaterial barrier administration.^[^ ^2^ ^]^ However, topical or systemic drug treatments both may cause side effects and trigger antibiotic resistance. Integrating multiple biological functions into one dressing is a promising evolution path for all‐period wound nursing. Such as bioadhesive dressings with barrier function, including anti‐fouling,^[^ ^3^ ^]^ antibacterial,^[^ ^4^ ^]^ and postoperative anti‐adhesion ability,^[^ ^5^ ^]^ have been developed to further fit clinical demand. However, under the premise of ensuring that dressings have excellent bioadhesive and postoperative protection performance, they still show limited clinical effects. On one side, it is crucial to develop a stable and durable barrier layer with a demand‐satisfying inertness interface, which is defined must relying on the surface environment of the target tissue. That varies considerably among tissue types.^[^ ^6^ ^]^ For example, a gastric ulcer staying in a physiological digestive environment needs dressing to isolate gastric juices and provide lubrication, just like the mucus‐bicarbonate barrier;^[^ ^7^ ^]^ skin as the first physical barrier once damaged, needs dressing to protect the body from the external environment, including microorganisms, dehydration and mechanical damage.^[^ ^8^ ^]^ Other side, operation accessibility corresponding to clinical settings should be considered rather than just meeting functional design requirements.^[^ ^6^, ^9^ ^]^ Because the wounds are mostly covered with body fluids or blood, dry and appropriately hydrophobic treated bioadhesives were created to break through the adhesion barrier from the interfacial water.^[^ ^10^ ^]^ For delivery convenience and full coverage of the wounds, in situ forming dressings, including assembly of powders and crosslinking of precursor solutions,^[^ ^5^, ^11^ ^]^ have emerged as attractive candidates. Predictably, ideal wound dressing should be multi‐barrier and accessible, not only close the wound to prevent body fluid loss and shield the external environment to avoid postoperative infection and adhesion but also possess delivery convenience and morphology adaption.

Reducing the interfacial interaction between dressings and tissues through hydrophobic design or inert functional group selection is an effective way to develop anti‐tissue adhesion materials. To fulfill the above requirements, we attempt to introduce slippery liquid‐infused porous surfaces (SLIPS) with dynamic liquid surfaces as barrier functions of dressing, because of its universally and efficiently anti‐biofouling properties.^[^ ^12^ ^]^ However, artificial adhesives for tissues are typically either hydrophilicity and strong interaction or hydrophobicity with limited strength, and tight fixation on the target tissue is the prerequisite for the dressing to function. Thus, we propose to integrate bioadhesive hydrogel and SLIPS into hybrid structures, named slippery‐adhesive Janus gel (SAJG), and in situ gelation strategy is adopted. Although there are related reports about the preparation of robustness of hydrogel‐elastomer hybrids by chemical bonding or mechanical anchoring.^[^ ^13^ ^]^ Considering the implementability of in‐situ preparation in vivo, these strategies are suboptimal. Here, we crosslinked the hydrogel and elastomer by mild and efficient silane coupling reaction, which only reacted in the interface of hydrogel‐elastomer. Because of the generally anti‐fouling ability of SLIPS, SAJG exhibits excellent performance of liquid repellency, anti‐tissue adhesion, antibacterial and protein adhesion. Further, rat models also showed excellent antibacterial infection and anti‐tissue adhesion properties of SAJG no less than corresponding functional hydrogels.

## 2 Results and Discussion

### 2.1 Design of SAJG

**Figure 1:**
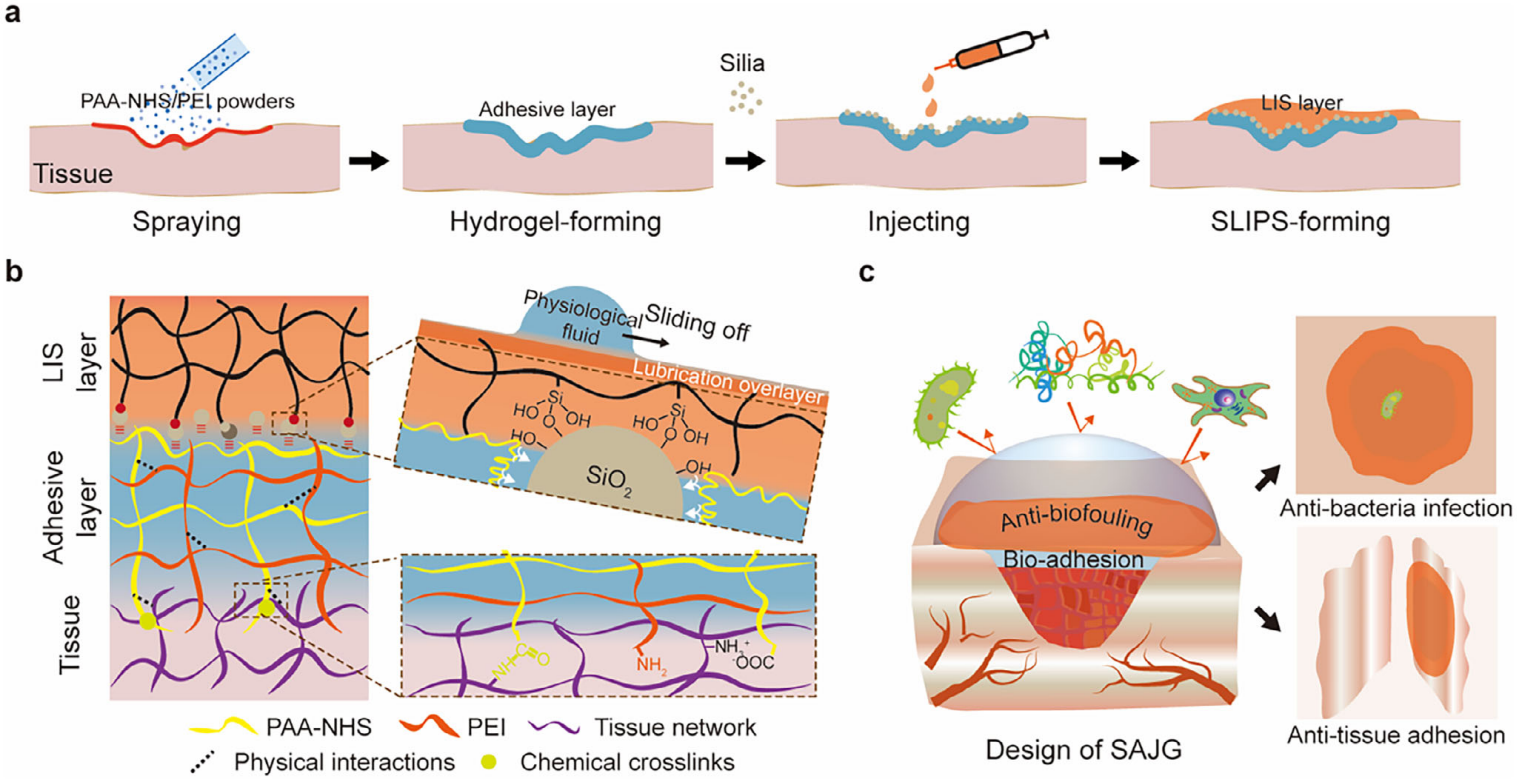
Design and properties of SAJG. a) SAJG was prepared layer by layer, including spraying powders, applying silica, and injection of precuring elastomer. b) SAJG consists of a hydrogel layer for adhering tissue and an LIS layer for providing anti‐fouling abilities. c) Schematic mechanism of SAJG to prevent bacterial infection and tissue adhesion.

SAJG shows extreme differences in wettability and bioadhesion on both sides, in which the powder‐formed hydrogel side is super‐hydrophilic with strong tissue adhesion and the liquid‐infused silicone (LIS) side is water‐repellent with anti‐adhesion effects (Figure S1, Supporting Information). SAJG is created through a straightforward procedure (**Figure** **1**a; Movie S1, Supplementary Movie). First, sprayable N‐hydroxysuccinimide grafted poly(acrylic acid)/ Polyethyleneimine (PAA‐NHS/PEI) powders rapidly absorb interfacial water to form a tough bioadhesive hydrogel. Subsequently, silica is applied onto the surface of hydrogel to adsorb onto polymer gels. Finally, pre‐polymerized silicone can be injected into the adhesive layer and establish a stable attachment during gelation, forming a slippery LIS layer. As shown in Figure 1b, the bottom adhesive layer crosslinks with tissue through physical interpenetration and chemical bonding, thereby ensuring strong and stable adhesion. Nano silica was introduced to enhance the interfacial strength of the LIS‐hydrogel because of its high specific surface area and extensive hydrogen bonding. The outmost LIS layer with lubrication overlayer consists of silicone polymer and silane coupling agent, which integrates into the silicone network to couple with the silica. The design of SAJG aims to exploit the robust bio‐adhesion properties of hydrogel for wound sealing while harnessing the anti‐biofouling ability of SLIPS to safeguard against postoperative infection and adhesion (Figure 1c).

### 2.2 Slippery Antiadhesion Properties of the LIS Layer

The LIS is prepared via a facile one‐step method by mixing polydimethylsiloxane (PDMS) precursor, silicone oil, and coupling reagent in a certain ratio. The gelation time of LIS was determined through manual manipulation and rheological analysis, indicating LIS forms within minutes (Figure S2, Supporting Information). In this system, both the content and viscosity of infused oil affect the slippery performance of LIS. Here, the 1:1 ratio of PDMS with silicone oil was determined. Diverse LISs were prepared by infusing different viscosity silicone oils, including 10, 1000, 1 × 10^4^ and 5 × 10^5^ cSt. Among them, LIS with 1000 cSt silicone is preferred to be characterized as LIS because of its excellent water‐repellent performance (Figure S1, Supporting Information).

**Figure 2:**
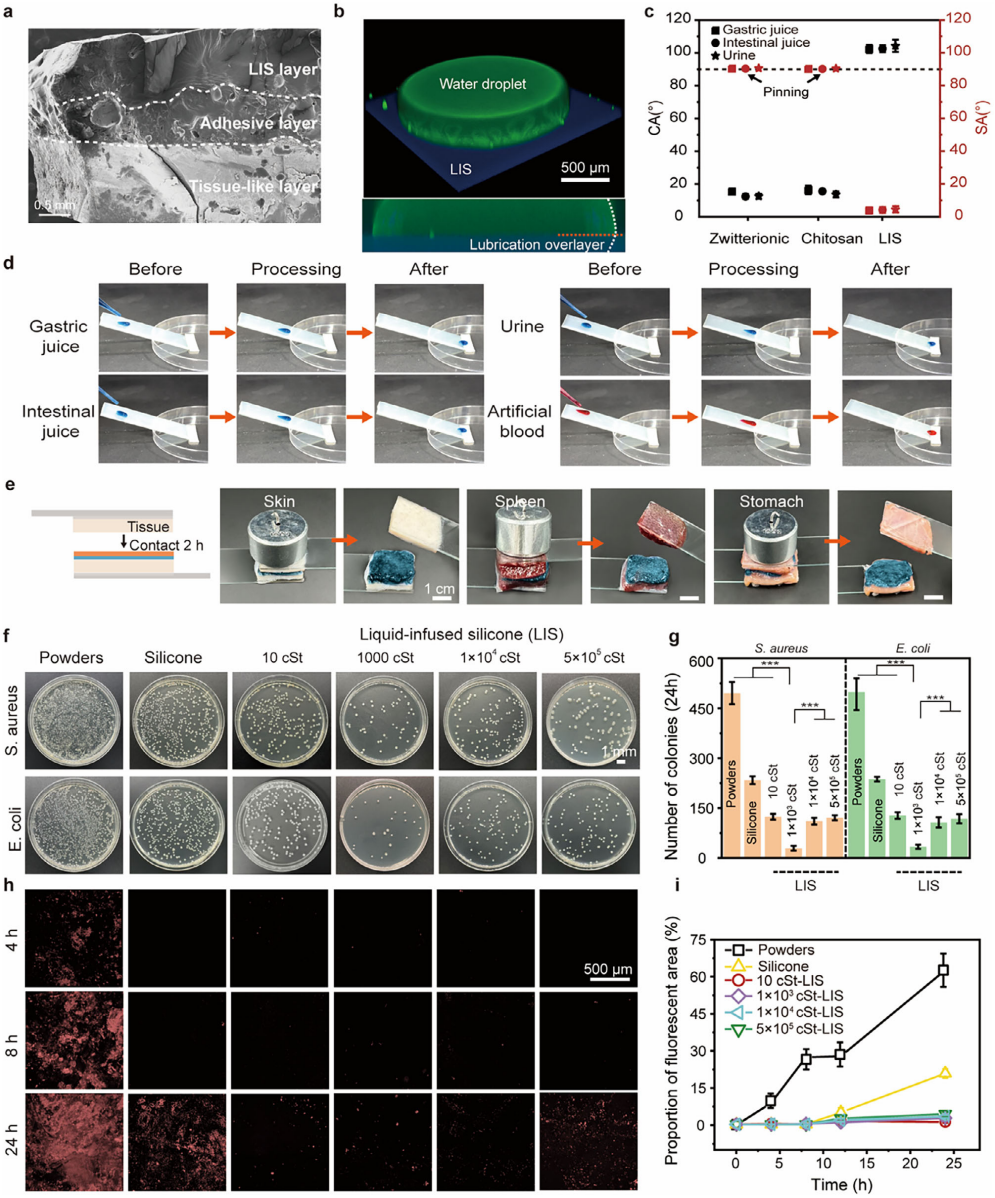
Lubrication and anti‐adhesion properties of LIS layer. a) SEM of SAJG shows its multilayered structure with tight integration. b) 3D confocal images of water droplets on the LIS. Overlapping color areas verify the existence of a lubrication overlayer. c) Contact angle and sliding angle of various physiological fluids on zwitterionic, chitosan hydrogel, and LIS. d) The process in which Fast Green FCF dyed physiological fluids slide over the surface of LIS. e) Anti‐tissue adhesion test of LIS layer. f) Optical images of antibacterial adhesion performance of hydrated powders, silicone, and LISs. g) Colony number of microorganisms formed on a solid medium. h) Confocal images of anti‐protein adhesion performance of hydrated powders, silicone, and LISs after 4, 8, and 24 h culture. i) The change of fluorescent areas over culture time on different surfaces.

The interlayer tightness and stability of LIS‐hydrogel play a vital role in the function of SAJG. The scanning electron microscope (SEM) observed that the multilayered structure of SAJG is tightly adhered without any gaps, demonstrating high structural adaptability and strong interlayer bonding (**Figure** **2**a). Relatively, the conjoint LIS‐hydrogel interface without silica will crack after freeze‐drying treatment (Figure S3, Supporting Information). The adhesion strength of LIS‐hydrogel with or without silica is shown in Figure S4 (Supporting Information). The excellent anti‐fouling performance of SLIPS is attributed to the presence of a thin lubrication overlayer on the surface of the silicone network. As shown in Figure 2b and Figure S5 (Supporting Information), the bottom of the green marked water droplet standing on a purple LIS platform exists a color overlap area, verifying the existence of the flexible lubrication overlayer. Compared with other anti‐tissue adhesion gels (zwitterionic, chitosan), LIS exhibited excellent hydrophobicity and was slippery for various physiological fluids, including gastric juice, intestinal juice, and urine, to protect wounds from corrosion (Figure 2c,d; Figure S6, Supporting Information). Zwitterion and chitosan gels were synthesized by referring to the methods described in previous studies.^[^ ^14^ ^]^ Besides, moist soil can be washed down by deionized water (Figure S7, Supporting Information). To verify the anti‐tissue adhesion performance of LIS, fresh tissues, protein, and bacteria as the testing medium were chosen. As shown in Figure 2e, a piece of cleaned tissue was covered on another piece of tissue applied SAJG. After two hours of waiting, compared to the control (Figure S8, Supporting Information), the lower tissue contacting the LIS layer cannot be lifted, indicating the effectivity of anti‐tissue adhesion. Staphylococcus aureus (S. aureus) and Escherichia coli (E. coli) were used for the antibacterial adhesion test. After 6 and 24 h bacteria attachment, the appendiculate bacteria of sample surfaces were centrifuged down, spread plate, and incubated overnight. Results show LIS series has remarkable antibacterial attachment properties compared with both hydrogel and hydrophobic silicone (Figure 2f,g; Figure S9, Supporting Information), which point straightly at interface wettability behavior. Further, fluorescein‐labeled bovine serum albumin (FITC‐BSA) was used in the anti‐protein adhesion test. After 4, 8, and 24 h protein attachment, the absorptive proteins on the sample surfaces were observed by confocal microscopy. In Figure 2h,i, the fluorescence area was positively correlated with the number of adsorbed proteins, proving the excellent anti‐protein adhesion ability of LIS.

### 2.3 Strong Tissue Adhesiveness of PAA‐NHS/PEI Powders

**Figure 3:**
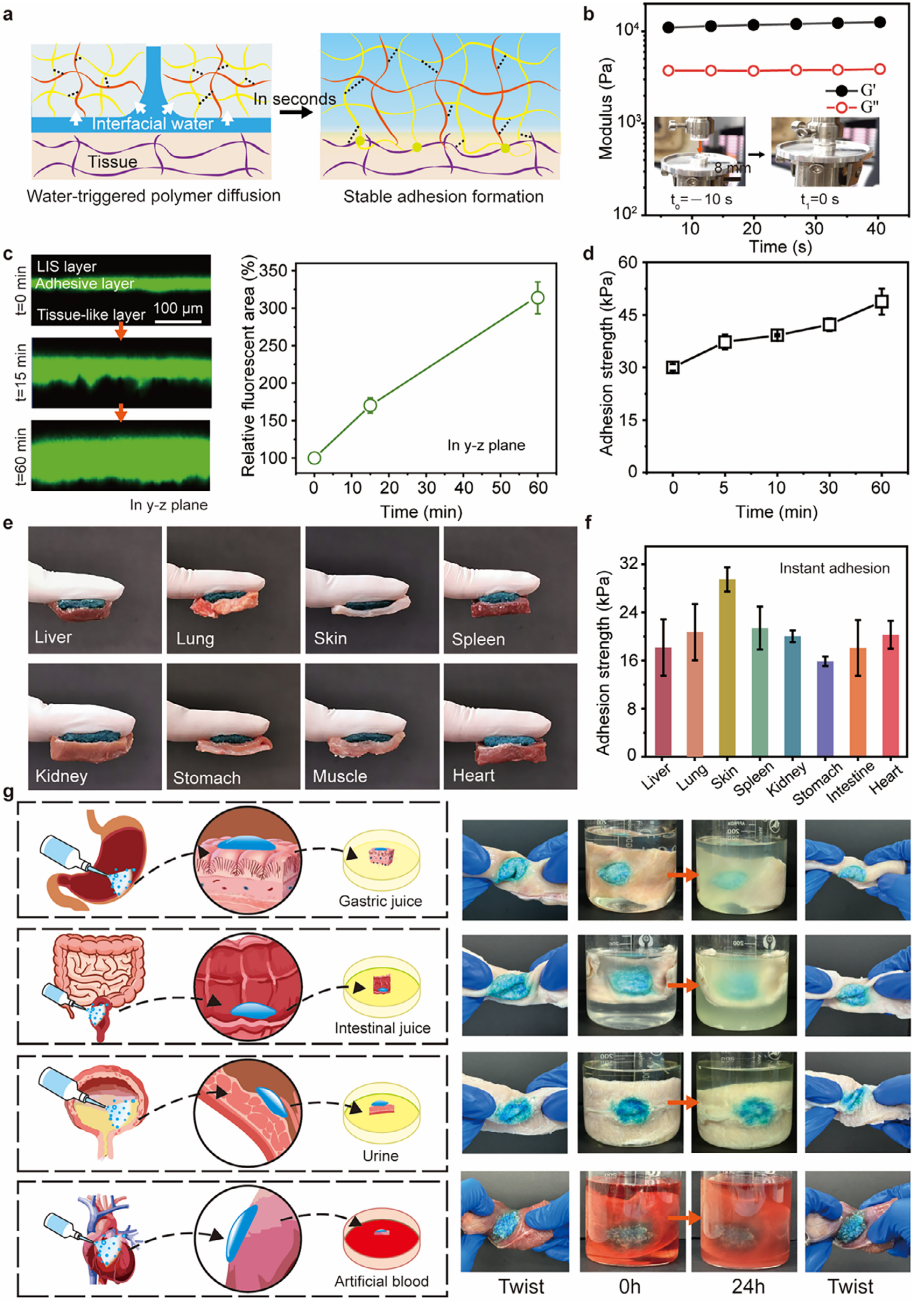
Hydration and wet adhesion properties of PAA‐NHS/PEI powders. a) Schematic illustration of hydration and adhesion of PAA‐NHS/PEI powders. b) Gelation time of PAA‐NHS/PEI powders once meeting the PBS buffer by rheological analysis. c) Confocal images of physical interpenetration of hydrated powders between the LIS layer and prefabricated PAM gel matrix, and the change of relative fluorescent area over time. d) Adhesion strength of hydrated powders to fresh porcine skin over time. e) Optical images of hydrated powders adhering to diverse tissues. f) Adhesion strength of hydrated powders to diverse tissues. g) PAA‐NHS/PEI powders were sprayed onto various porcine tissues and subsequently immersed in the corresponding physiological fluids for 24 hours, manifesting favorable interface compatibility and stability.

PAA‐NHS/PEI powders can quickly absorb the interfacial water of tissue and induce polymer diffusion, forming hydrogel and stitching hydrogel tissue together (**Figure** **3**a). NHS grafted on PAA aims to couple free amine‐containing biomolecules of tissue network to enhance adhesive‐tissue interaction. Compared to pure PAA, the infrared spectra of PAA‐NHS with high NHS ester showed associated triplex bands at 1730, 1780, and 1815 cm^−1^ of NHS were detected and the signal of acid with a single band at 1705 cm^−1^ was obviously weakened, verifying the formation of PAA‐NHS (Figure S10, Supporting Information).^[^ ^15^ ^]^ Although the NHS group of PAA‐NHS may amidate with PEI's ‐NH_2_, NHS did not fully react and remained on the surface of the material (Figure S11, Supporting Information). Moreover, the augmentation of NHS grafting rate is accompanied by a concomitant increase in immediate adhesion strength to porcine skin (Figure S12, Supporting Information), underscoring the indispensability of NHS for activating carboxyl groups and augmenting tissue adhesion. Rheological tests and optical micrographs show the gelation formation of the PAA‐NHS/PEI powders within seconds once exposed to phosphate buffer saline (PBS) in Figure 3b and Figure S13 (Supporting Information). It should be noted that it takes about 5 s for the powders (1 mm thickness) fixed on the top die to come into contact with the arch droplet (100 µL) before starting the test (t_1_ = 0 s). Obtained PAA‐NHS/PEI powders were divided into different sizes using sample sieves. Results show the small powder size is conducive to excellent adhesion (Figure S14, Supporting Information). Further, fluorescently labeled powders were prepared by labeling PAA polymer, which was obtained by copolymerizing fluorescent monomer and acrylic monomer.^[^ ^14a^ ^]^ The gelation and adhesion behavior of powders were observed by confocal laser scanning microscopy (CLSM). Fluorescence images and statistical data revealed that the fluorescence areas of the initial hydrous powders exhibited dispersion and gradual increase over time (Figure S15, Supporting Information), indicating the progressive maturation of the hydrogel network upon macroscopic hydrogel matrix formation. Especially, interlayer polymer diffusion of powder‐formed hydrogel on both LIS and tissue‐like sides (Figure 3c) revealed that the PAA‐NHS/PEI hydrogel diffused into the tissue‐like layer (polyacrylamide (PAM) hydrogel^[^ ^16^ ^]^), as evidenced by the increasing fluorescent area downward. In contrast, there was no significant penetration at the LIS‐hydrogel interface due to pronounced molecular polarity differences between them. The adhesion strength of the powder‐formed hydrogel to porcine skin also exhibits positive time‐dependence and demonstrates strong tissue adhesion (Figure 3d). PAA‐NHS/PEI powder gel exhibits remarkable adhesive properties as it swiftly and firmly adheres to a variety of tissues, including the liver, lungs, skin, spleen, kidney, stomach, muscle, and heart (Figure 3e,f). Besides this, the PAA‐NHS/PEI hydrogel manifested favorable interface compatibility, keeping a low swelling ratio and excellent adhesion stability in a variety of humoral physiological fluid environments after 24 h soak (Figure 3g; Figure S16, Supporting Information). Meantime, in vitro adhesion strength test of hydrated PAA‐NHS/PEI powders after immersing in the above fluid environments was collected, indicating >10 kPa adhesion strength after 7 h immersion (Figure S17, Supporting Information).

### 2.4 In Vivo Antibacterial and Wound Healing Ability of SAJG

**Figure 4:**
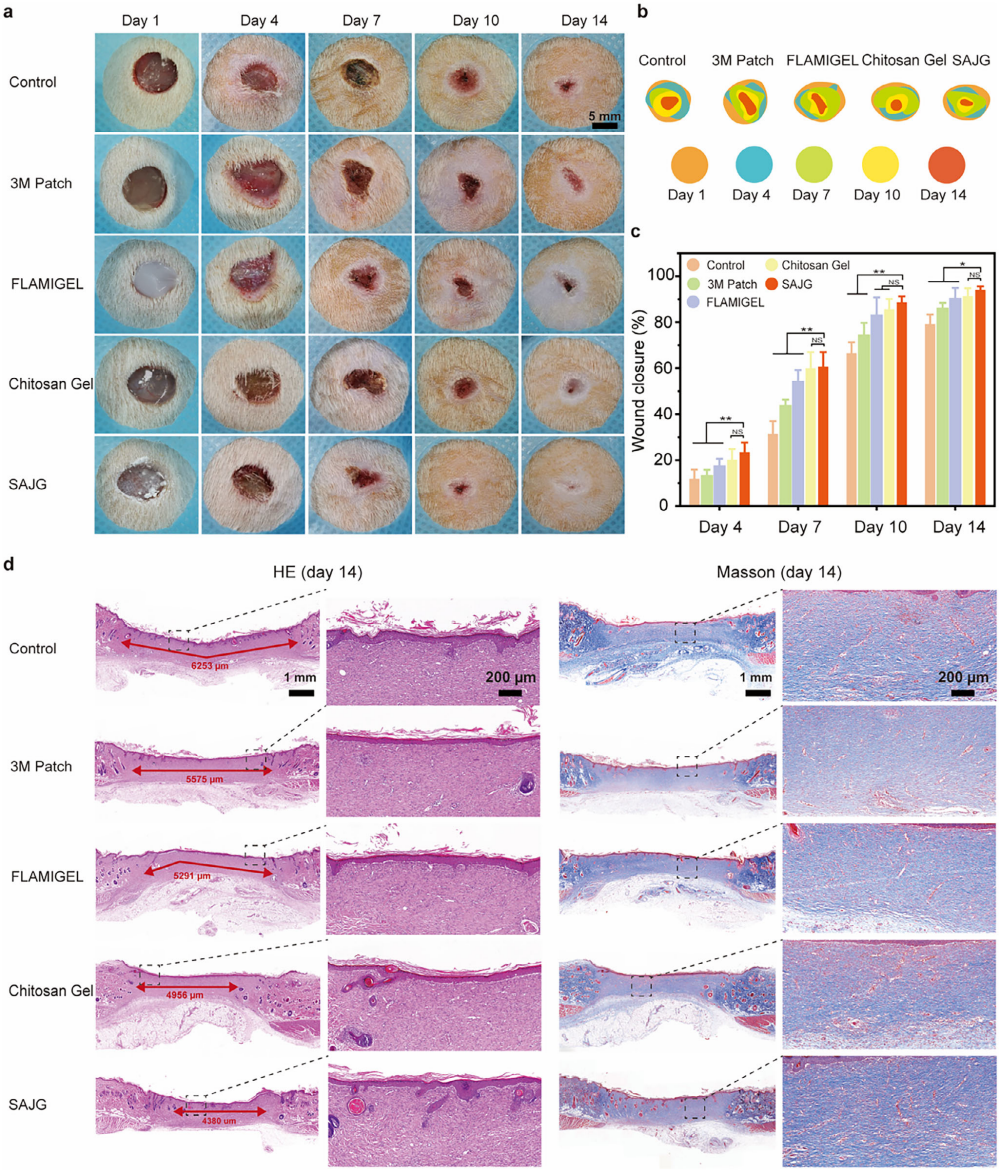
Accelerating healing ability of SAJG. a) Representative images of infected wounds treated with different dressings at different periods. b) Diagram of the dynamic wound healing process, and c) quantitative analysis of wound area (*n* = 5) within 14 days for each treatment. d) H&E and Masson staining during the wound healing process.

Before in vivo experiments in live animals, we performed cytotoxicity assays. The in vitro biocompatibility of the SAJG is detected by L929 cell culture. Comparable to the control group, SAJG demonstrated comparable cell activity, indicating its excellent biocompatibility (Figure S18, Supporting Information). To evaluate the antibacterial ability of SAJG, we constructed the skin infection model of rats (Figure S19a, Supporting Information). Commercial 3 M Patch, FLAMIGEL, Chitosan Gel, or SAJG or no treatment (as a control group) were covered on the wound. Then, S. aureus suspension was added to the center of the wound. The Spread plate method is chosen to evaluate the antibacterial ability. Optical images and colony count indicate that FLAMIGEL, Chitosan Gel, or SAJG have an obvious bacteriostatic effect, especially SAJG (Figure S19b, Supporting Information). The antibacterial properties of FLAMIGEL and Chitosan Gel are mainly derived from the cationic polymers (arginine or chitosan) they contain.^[^ ^17^ ^]^ Differently, the antibacterial effect of SAJG derives from the lubrication layer having low bacteria adhesion and is not conducive to bacteria survival. Representative images and statistics of wound healing (**Figure** **4**a,b) also show the swiftest healing progression of the SAJG group among all treatment cohorts, achieving a final wound closure of 94% (Figure 4c). To gain a more nuanced understanding of the healing process, pathological staining was performed (Figure 4d). The pathologist remained unaware of the grouping of the animals when analyzing the tissue sections of the animals. Hemotoxylin and eosin (H&E) staining results underscored the accelerated wound contraction observed in the SAJG group, with a notably smaller wound area, which was consistent with the macroscopic photographic results. Moreover, the epidermis in the SAJG‐treated wounds appeared more intact, accompanied by the emergence of appendages like hair follicles, indicative of a robust regenerative process. Masson's trichrome revealed a denser and more organized collagen deposition pattern in the SAJG groups (Figure S20, Supporting Information), suggesting tissue remodeling and maturation during the healing process.

### 2.5 In Vivo Postoperative Adhesion of SAJG

**Figure 5:**
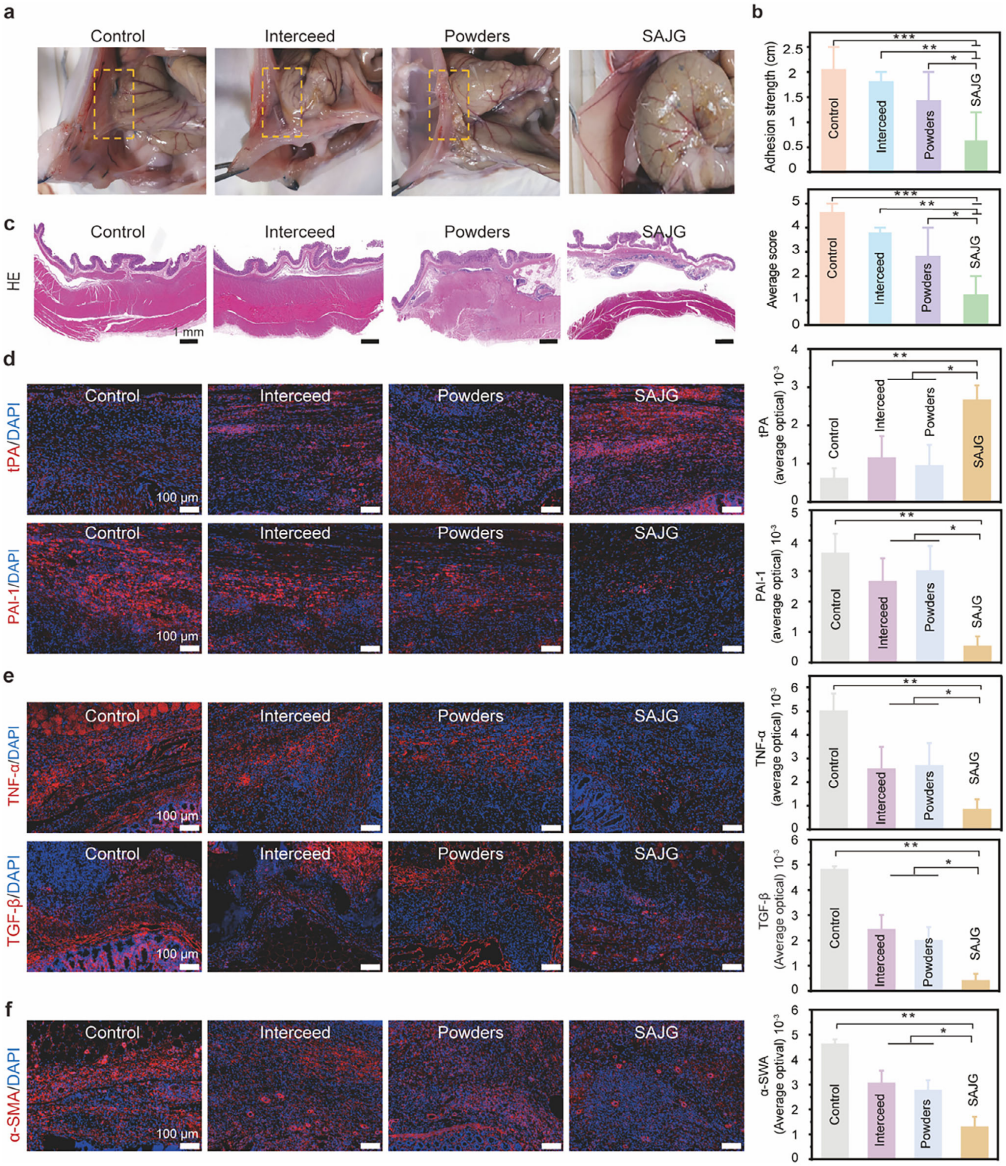
Anti‐adhesion efficiency of SAJG in a murine cecum‐abdominal wall adhesion model. a) Typical abdominal adhesions in the Control, Interceed, PAA‐NHS/PEI powders, and SAJG 14 days after operation. Yellow dashed boxes indicate the adhesion sites. b) Adhesion scores and adhesion length. c) Typical H&E staining images of tissue sections in different groups. d–f) Typical immunofluorescence staining images and immunofluorescence signal intensity of tissue sections in different groups.

To evaluate the antiadhesion ability of SAJG, we constructed the intestine damage model of rats (Figure S21, Supporting Information). The damage with about 3 cm length was created along the trend of the intestine and the other side was created on the contralateral abdominal wall with the corresponding area. After surgery, the damaged area was covered with INTERCEED (an absorbable fabric designed to reduce the formation of postsurgical adhesions), PAA‐NHS/PEI powders, SAJG, and suture the damages. In the control group, damages were not treated before suture. After 2 weeks, rats were euthanized and evaluated for the adhesion degree. After intestine damage, no rats showed abnormalities in survival state, appetite, or sleep status during the observation period. As shown in **Figure** **5**a, severe adhesions occurred between the abdominal muscle layer and the cecum in the control group (marked by yellow dashed boxes). According to the classic clinical scoring system, the average adhesion scores were given from 0 to 5, 0 indicating no adhesion and 5 indicating very dense and vascularized adhesion.^[^ ^18^ ^]^ In the control group, the noticeable adherence between the cecum and the abdominal wall was recorded, exhibiting an average adhesion strength of 2 cm and an average score of 4.5 (Figure 5b). Compared to the control group, all covered groups exhibited a reduction in the severity of tissue adhesion to a certain extent. Notably, SAJG showed great advantages in preventing tissue adhesion. H&E staining and Masson's trichrome staining provided histological visualization of the tissue adhesions and healing processes at the injured sites (Figure 5c; Figure S22, Supporting Information). The control group had accumulated connective tissues accompanied by a large number of leukocytes, proving that the formation of the adhesions was closely related to inflammation.^[^ ^19^ ^]^ The PAA‐NHS/PEI powders and commercial INTERCEED groups displayed moderate adhesions, featuring slight connections or bridges between the cecum and the adjacent abdominal wall. In contrast, there was no apparent adhesion between the abdominal wall and the cecum after treatment with SAJG. The application of Masson's trichrome staining revealed a significant accumulation of collagen in the control group. While the commercial INTERCEED and PAA‐NHS/PEI powders exhibited some protective effects, collagen deposition was still relatively obvious, leading to the formation of mild to moderate adhesions. In line with the findings from the H&E staining, the SAJG group exhibited minimal to no collagen deposition, showcasing its outstanding antiadhesive properties.

Previous investigations have highlighted the pivotal role of the imbalance between fiber degradation and deposition in triggering the development of postoperative adhesions.^[^ ^20^ ^]^ Tissue plasminogen activator (tPA), a critical mediator of fibrinolysis, faces opposition from plasminogen activator inhibitor (PAI)‐1, which reduces fibrinolytic activity.^[^ ^21^ ^]^ We thus further evaluated the expressions of tPA and PAI‐1 in the injured tissues 14 days after the operation (Figure 5d). The expression of the antifibrinolytic PAI‐1 in the SAJG group is diminished while the pro‐fibrinolytic tPA has the opposite result. These findings seem to imply the presence of a more balanced fibrinolytic environment within this group. In addition to their roles in fibrinolysis, we also assessed other important mesothelial and fibroblast markers in the abdominal adhesions, namely tumor necrosis factor‐α (TNF‐α), transforming growth factor‐β (TGF‐β), and α‐smooth muscle actin (α‐SMA).^[^ ^22^ ^]^ Our results indicated that the SAJG group exhibited a lower immunofluorescence signal intensity for both TNF‐α and TGF‐β compared to the control and commercial adhesion barrier groups (Figure 5e). The fibrotic scars present at abdominal adhesion sites, where the abdominal wall adheres to visceral layers, were characterized by an upregulation of α‐SMA expression. In the control group, the expression of α‐SMA was pronounced at the site of adhesion, whereas the α‐SMA expressions were reduced after the treatment with the SAJG and Interceed, with the SAJG group exhibiting the most pronounced decrease (Figure 5f).

## 3 Conclusion

Postoperative infection and adhesion present significant challenges in wound management. In this work, we utilized the slippery anti‐fouling properties of dynamic liquid‐infused surfaces and developed an in‐situ forming slippery‐adhesive Janus gel (SAJG) suitable for the repair and postoperative nursing of wounds. SAJG, which was composed of an adhesive hydrogel layer and a slippery liquid‐infused silicone (LIS) layer, was created layer by layer, achieving both strong tissue adhesion and antiadhesion ability. PAA‐NHS/PEI powders with excellent wet tissue adhesion (about 30 kPa instant adhesion to porcine skin) construct a hydrogel layer by absorbing the interfacial water. LIS was created by a one‐step injection on the hydrogel layer. Compared to commercial bioactive dressing, SAJG without active ingredients indicated high antibacterial efficiency and anti‐tissue adhesion ability, which was verified by both in vivo and in vitro tests. We believe that the dynamic lubrication layer strategy is promising for postoperative wound management and the mechanism of preventing tissue adhesion needs to be further studied.

## 4 Experimental Section

### Preparation of PAA‐NHS/PEI Powders

To achieve strong and stable adherence to wet tissues, PAA‐NHS/PEI powders are prepared by reference to previous reports.^[^ ^23^ ^]^ First, 1 g of the PAA (Mw, ca. 240000) was added into 8 g dichloromethane (DCM) / dimethylformamide (DMF) (1:1) and stirred overnight to obtain solution A. Then, 1‐ethyl‐3‐(3‐dimethylaminopropyl) carbodiimide (EDC) and N‐Hydroxysuccinimide (NHS) (the molar ratio of EDC/NHS was 1:1; the molar ratios of NHS/COOH were 1:1, 1:3 and 1:7.) were pre‐dissolved separately in DCM/DMF (1:1), named B and C. Solution B was added dropwise to the A and stirred for 5 min, then solution C was added in the same manner and stirred for 1 h under ice cooling. After that, the reaction mixture was further stirred at room temperature for 12 h. During the reaction, PAA‐NHS precipitates in the reaction mixture. The obtained product was subsequently purified by washing several times with acetone and then dried under a vacuum. We mixed 10 wt.% PAA‐NHS and 10 wt.% PEI (Mw, ca. 70000) aqueous solutions in the following volumetric ratios of 2:1. The mixtures were immediately immersed in liquid nitrogen, and then freeze‐dried to remove water. Last, the dried solids were ground to obtain PAA‐NHS/PEI powders using a mortar and pestle. The size range of powders was regulated by a sifter. Fast Green FCF was physically mixed into the PEI or PAA‐NHS aqueous solution to prepare green powders.

### Preparation of LISs

The LIS is prepared via a facile one‐step method by mixing Ecoflex 00–35 Fast (two‐component platinum‐catalyzed silicones), silicone oil, and vinyltrimethoxysilane (VTMS). First, part A and part B of Ecoflex 00–35 Fast (freeze to −20 °C before use) following 1:1 were poured into a clean polyethylene plastic cup and quickly stirred to mix. Then a certain amount of silicone oil and 0.1 wt.% VTMS was added in sequence at room temperature. The mixture is centrifugated 90 s at 2000 r min^−1^ to degas before use. The whole process needs to be done quickly to keep the mixture at a low temperature.

### Lap Shear Adhesion Test

In the lap shear adhesion assessment, two pieces of biological tissue specimens, each precisely dimensioned at 2.5 cm in length (L) and 1.5 cm in width (W) were cut using a scalpel. Subsequently, one specimen was covered with a 500‐micrometer‐thick layer of PAA‐NHS/PEI powders, and the other second specimen was sprayed with PBS buffer solution. By contacting these prepared specimens, a junction contact area of 1.5 cm^2^ was formed. Gentle finger pressure was applied to the lap joint for approximately 5 s to ensure stable contact. Following this, the specimen assembly underwent a shear adhesive test to evaluate the adhesive bond formed between the tissues, using a tensile testing machine (INSTRON 5943) at a strain rate of 20 mm min^−1^. The adhesion strength was calculated as follows: adhesion strength = F_max_/ (L × W).

### Antitissue Adhesion Test

To analyze the anti‐adhesive properties of SAJG, standardized 2.5 by 2.5 cm segments of varied biological tissues (comprising skin, stomach, and spleen) were fixed to glass slides, and subsequently, SAJG was applied to these tissues. For comparison, control groups consisted of tissues solely covered with PAA‐NHS/PEI hydrogel. Then, fresh tissues were pressed on the SAJG or the hydrogel (with 100 g weight placed on the top). Following a 2 h waiting period, the adhesion status of each group was observed and recorded.

### Antibacterial and Anti‐Protein Attachment Test

S. aureus (ATCC 6538) and E. coli (ATCC 25922) were used in this antibacterial test. The model bacteria were resuscitated, propagated, diluted by gradient, and measured with a microplate reader to obtain a bacterial suspension at a concentration of ≈10^7^ CFU mL^−1^. Samples were placed in 24‐well plates, which contained 2 mL LB liquid culture medium with bacteria (10^7^ CFU mL^−1^). The plates were kept at 37 °C for different times of incubation (6 and 24 h) with a table concentrator. The samples were collected and gently rinsed with PBS buffer to remove free bacteria on the surface. Then rinsed samples were placed into a 1.5 mL centrifuge tube filled with PBS buffer, then centrifuged at 8000 rpm for 5 min to make colonizing bacteria off. The LB nutrient agar plates were uniformly spread with 50 µL of the centrifuged solution, subsequently cultured overnight, and then subjected to counting procedures. FITC‐BSA was used for the anti‐protein adhesion assay. 10 µL FITC‐BSA was added to 20 mL PBS solution to obtain the protein suspension, and the formed samples were placed in it and incubated at 37 °C for different times of incubation (4, 8, and 24 h). Finally, the samples were collected and gently rinsed several times with PBS buffer to remove free proteins on the surface. Confocal microscopy was used to observe the protein adhesion on the surface of the samples.

### Rheological Characterization of Powders and LISs

DHR‐3 rheometer (TA Instruments) with 8 mm diameter stainless steel parallel plates was used for rheology tests of powders. The samples were prepared by mixing PAA‐NHS/PEI powders with PBS. The parallel plate gap was set to 1100 µm. The frequency sweep test was performed on the hydrogels at a strain of 0.1% at room temperature, and frequency from 0.1 to 10 Hz. To characterize the gelation rate, the PAA‐NHS/PEI powders were attached to the upper plate by double‐sided tape and then contacted with the PBS solution located on the lower plate. Once the designated location was reached, a time scan was started to determine the modulus change of the hydrogel, under a fixed strain of 1.0% and a frequency of 1 Hz at room temperature. Gelation time was defined as the time point when the storage modulus of the PAA‐NHS/PEI hydrogel exceeded the loss modulus.

Dynamic viscoelasticity of the LISs was measured using Haake Mars 40 (Thermofisher Scientific) with 20 mm stainless steel parallel plates. The samples were prepared by mixing Ecoflex 00–35 Fast (two‐component platinum‐catalyzed silicones), silicone oil and VTMS. The oscillatory time sweep experiments were conducted under a fixed strain of 1.0% and a frequency of 1 Hz at 37 °C.

### Gelation Time Test

The gelation time of LISs was measured manually: the temperature of the water bath was adjusted to 25 ± 0.5 °C. Component A, VTMS, and silicone oil were weighed with a pharmaceutical balance using a beaker as a container to achieve an accuracy of ±0.2 g. Place the beaker in a water bath (with the liquid level 2 mm below the water surface) at constant temperature and stir carefully. When the sample temperature reached 25 ± 0.5 °C, component B was added, the stopwatch was started and the sample was stirred. Observe the sample every 30 s, and test the flowability with a glass rod until a string‐forming state appears. Record the time indicated on the stopwatch, which is the gelation time.

### Cell Culture Experiment

The assessment of hydrogel cytocompatibility was conducted using two approaches: Live/Dead imaging and cell counting kit‐8 (MCE, HY‐K0301, CCK8) assay. L929 fibroblasts (ATCC) were seeded into a 96‐well plate at a density of 1 × 10^4^ cells per well, and cultured overnight in Dulbecco's Modified Eagle's Medium (DMEM, Gibco) culture medium containing 10% (v/v) fetal bovine serum (FBS, Gibco) and 1% (v/v) penicillin−streptomycin solution (P/S, Gibco). Subsequently, the existing medium was substituted with fresh medium containing PAA‐NHS/PEI (1 or 5 mg mL^−1^) hydrogel or SAJG (1 or 5 mg mL^−1^). The samples were then subjected to further cultured at 37 °C in a humidified incubator containing 5% CO_2_ for 3 days. Following this incubation period, the cells were stained with 500 µL of calcein‐AM/propidium iodide dyes (Beyotime, C2015Mm) for 30 min. Subsequently, these cells were visualized and analyzed using an inverted microscope (Olympus, iX73) to show the green (live cells) and red (dead cells) fluorescence. The proliferation of L929 cells in the presence of PAA‐NHS/PEI hydrogels and SAJG hydrogels was assessed utilizing the standardized culturing protocol in conjunction with the CCK8 assay (MCE). Following 24 and 72 h of incubation, the existing medium was discarded, and the cells were gently rinsed with PBS. Subsequently, 100 µL of complete medium supplemented with 10 µL of CCK8 reagent was dispensed into each well, followed by incubation for 1 h. The optical density at 460 nm (OD460) of each well was measured using a microplate reader (BioTek, MQX200). The untreated cells incubated in a standard culture medium were designated as a control.

### In Vivo Antibacterial Models of Rat

The experimental protocols were rigorously reviewed and approved by the Experimental Animal Ethics Committee of West China Medical College following the Institutional Guidelines (202407260002). Four‐week‐old male Sprague‐Dawley (SD) rats weighing approximately 200–250 g were used in this experiment. Prior to experimentation, the rats were anesthetized through inhalation of 3% isoflurane. The rats underwent hair removal from their backs using a combination of electric shaver and hair removal cream to prepare the skin surface. Subsequently, five circular full‐thickness skin defects, each with a diameter of 10 mm, were created on each rat's back using a dedicated skin biopsy instrument. Immediately after, dressings, including TegadermTM Hydrocolloid Thin Dressing (3M Patch), Flamigel Wound gel dressing (FLAMIGEL), RMC Chitosan Medical Bio‐gel (Chitosan Gel) and SAJG, were gently applied over the wounds. A bacterial suspension of S. aureus at a concentration of 10^7^ CFU mL^−1^ was then dripped on each dressing site in a volume of 100 µL. The control group remained untreated throughout the study period. The quantitative assessment of wound area reduction was performed using Image J software (NIH, USA). The percentage of wound closure was calculated employing the formula: Wound closure (%) = (1‐Wound area_t_/Wound area_0_)×100%, where the Wound area_t_ and Wound area_0_ represent the wound areas measured on day_t_ and day_0_, respectively. To further quantitatively analyze the in vivo antibacterial efficacy, bacteria samples were isolated from the collected wound tissue and subsequently cultured on LB nutrient agar plates. Subsequently, a quantitative analysis was conducted.

### In Vivo Antipostoperative Adhesion Models of Rat

The animal experiment was ethically approved by the Animal Experimentation Ethics Committee of West China Medical College (202407260001). Male SD rats, weighing between 200 and 250 g, were utilized to establish postoperative adhesion models. Prior to surgery, the rats were anesthetized using a 1.5% sodium pentobarbital solution in conjunction with oxygen inhalation support to ensure stable and comfortable physical status. To establish the intestinal adhesion model, the cecum and adjacent abdominal wall were scraped with gauze and tweezers until the epidermis was damaged. Following this, various barriers were applied to the injured cecum (Control vs Interceed vs PAA‐NHS/PEI powders vs SAJG). Postoperatively, the ends of the injured cecum and abdominal wall were sutured with 6‐0 sutures, ensuring its stable positioning for the development of adhesions. After skin dissection, the sutured line was reopened to expose the injured/repaired site to evaluate the results of the adhesion formation and injured tissue healing. To objectively quantify the extent of adhesions in the rats, a rigorous double‐blind scoring approach was employed, adhering to a standardized adhesion scoring system. The system entailed the following categories: score 0, no adhesion; score 1, one thin filmy adhesion that requires gentle blunt dissection to be divided; score 2, more than one thin adhesion; score 3, one thick adhesion with a focal point; score 4, one thick adhesion with plantar attachment or more than one thick adhesion with focal points; score 5, very thick vascularized adhesions or more than one plantar adhesions. Upon completion of the experimental period, rats were euthanized, and the collected tissues were fixed in a 4% (w/v) formaldehyde solution, paraffin‐embedded, and sectioned. H&E staining and Masson's trichrome staining of sections were conducted using standard methods. Additionally, immunofluorescence staining was performed to detect specific biomarkers within the tissue sections. The intensity of the immunofluorescence signals was quantitatively assessed using Image‐pro plus 6.0 (Media Cybernetics, Inc., Rockville, MD, USA).

## Conflict of Interest

All authors have no conflict of interest to disclose.

## Author Contributions

Y.G., J.G., and S.L. contributed equally to this work. X.D. and B.H. conceived the idea and supervised the project. Y.G. and J.G. designed the study. S.L. and Y.Z. contributed to the preparation of materials. S.L. conducted the wettability test, lap shear adhesion test, and anti‐protein adhesion test. Y.G. and J.L. conducted the antibacterial experiment. Y.G. and J.G. conducted the rheological experiments. Y.G., L.C., and T.P. conducted the biocompatibility experiments. Y.G., L.Y., B.L., and J.G. conducted the animal experiments. Y.G., J.G., and S.L. drafted the manuscript. J.L., J.Y., D.W., C.X., B.H., and X.D. revised the manuscript. All authors reviewed and approved the final manuscript.

## Supporting information



Supporting Information

Supplementary Movie

## Data Availability

The data that support the findings of this study are available on request from the corresponding author. The data is not publicly available due to privacy or ethical restrictions.
