# Supplementary material for: A Bio‐Adaptive Janus‐Adhesive Dressing with Dynamic Lubrication Overlayer for Prevention of Postoperative Infection and Adhesion
Source: Adv Sci (Weinh). 2025 Mar 20;12(18):2500138. doi: 10.1002/advs.202500138 (PMC12079332; doi:10.1002/advs.202500138)
Supplement: Supplementary file 1 — Supporting Information [file ADVS-12-2500138-s001.docx]

**Supporting Information for**

**A bio-adaptive Janus-adhesive dressing with dynamic lubrication overlayer for prevention of postoperative adhesion and infection**

Yuan Gao, Junchang Guo, Shuangyang Li, Liansong Ye, Binyang Lu, Jiaxin Liu, Jing Luo, Yijia Zhu, Liuxiang Chen, Tingfa Peng, Jinlong Yang, Dehui Wang, Chaoming Xie, Xu Deng, Bing Hu

**Experimental Materials**

All reagents were purchased and used without further purification. Polyethyleneimine (PEI), dichloromethane (DCM), dimethylformamide (DMF), N-Hydroxysuccinimide (NHS), acetone, Fast Green FCF, vinyltrimethoxysilane (VTMS), acrylic acid (AA), chitosan, N-N’-Methylenebis(acrylamide) and Rhodamine B were purchased from Aladdin Biochemical Technology Co., Ltd. Different viscosities of silicone oil were available from different manufacturers: Merck KGaA (10cSt ), Dow Corning (1000 cSt ), Aladdin Biochemical Technology Co., Ltd (1×10^4^ cSt), Beijing Hagibis Technology Co., Ltd (5×10^5^ cSt). Polyacrylic acid (PAA) was purchased from J&K Scientific Co., Ltd. Photoinitiator I-2959 was purchased from Merck KGaA. Ammonium Persulfate was purchased from Shanghai Titan Scientific Co., Ltd. 1-(3-Dimethylaminopropyl)-3-ethylcarbodiimide (EDC), N-(2-Amino-2-oxoethyl)acrylamide and [2-(Methacryloyloxy)ethyl]dimethyl-(3-sulfopropyl)ammonium Hydroxide were purchased from Macklin Biochemical Technology Co., Ltd. Ecoflex 00-35 Fast was purchased from Smooth-On Co., Ltd. Nano silica was purchased from Evonik Industries AG, micro silica was purchased from Jiangsu Huimai Powder Technology Co., LTD. LB nutrient agar was purchased from Solarbio Science & Technology Co., Ltd. Artificial blood was purchased from Phygene Bio-Technology Co., Ltd. Simulated gastric juice, small bowel, colonic fluid, bile and pancreatic juice were purchased from Hongfeng Technology Co., Ltd. Dulbecco’s Modified Eagle’s Medium (DMEM), fetal bovine serum (FBS) and penicillin−streptomycin solution (P/S) were purchased from Gibco Life Sciences (Invitrogen) Co., Ltd. Calcein-AM/propidium iodide dyeing (C2015M) was purchased from Beyotime Biotechnology Co., Ltd. CCK8 assay was purchased from MedChemExpress (MCE) Biotechnology Co., Ltd. Immunofluorescence antibodies were purchased from Saiweier Biotechnology Co., Ltd. Tegaderm^TM^ Hydrocolloid Thin Dressing (3M Patch) was purchased from Minnesota Mining and Manufacturing Co., Ltd. Flamigel^®^ Wound gel dressing (FLAMIGEL) was purchased from Flen Health Co., Ltd. RMC^®^ Chitosan Medical Bio-gel (Chitosan Gel) was purchased from Humanwell Healthcare (Group) Co., Ltd. Interceed anti-adhesion membrane was purchased from Ethicon LCC.


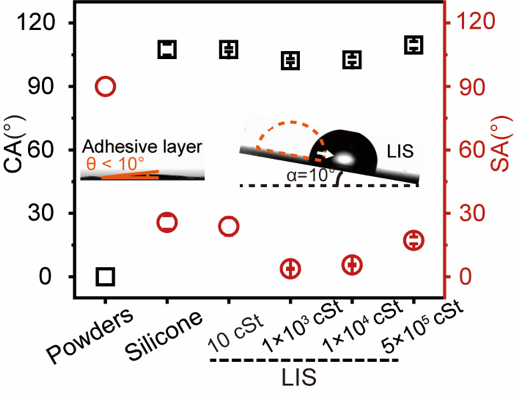


**Figure S1** The wettability of SAJG is characterized by contact angle and sliding angle, indicating liquid repellency on LIS side and superhydrophilicity on hydrogel side.


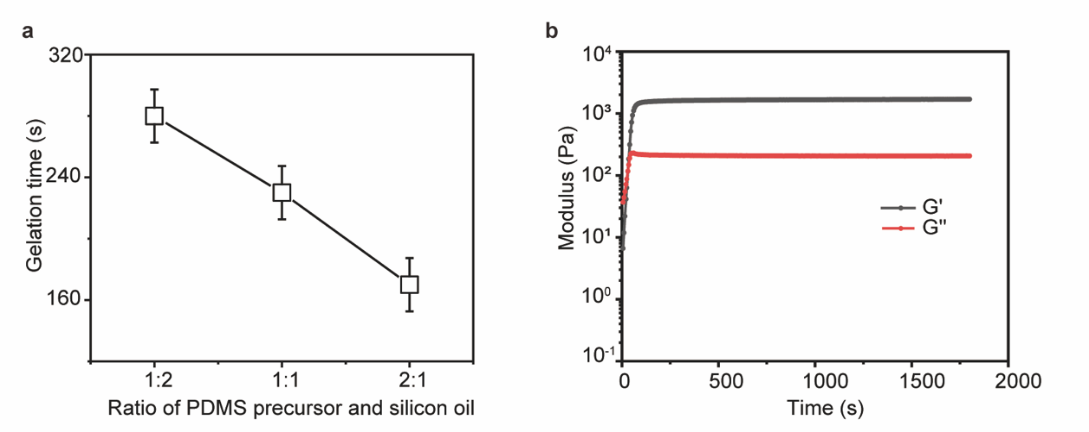


**Figure S2** a) Gelation time of LISs with different ratio of PDMS precursor and silicon oil as determined by manual operation. b) The oscillatory time sweep scan of 1:1 PDMS and 1000 cSt silicone oil.


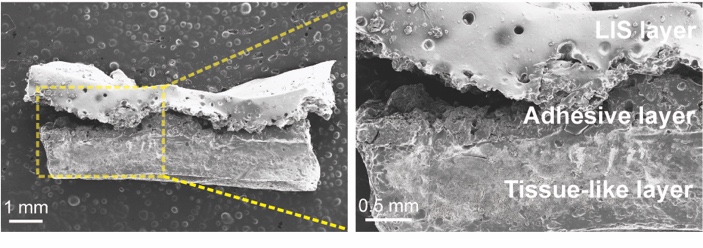


**Figure S3** Scanning electron microscopy (SEM) of SAJG without silica application. The crack happened between LIS layer and adhesion layer after freeze-drying.


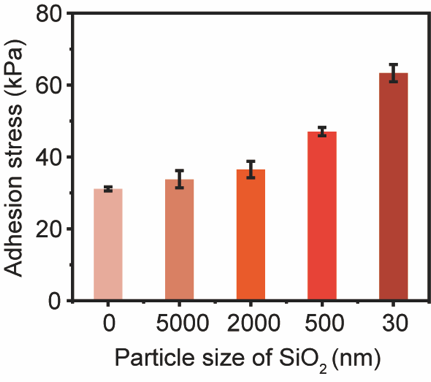


**Figure S4** The adhesion strength of LIS-hydrogel with or without silica.


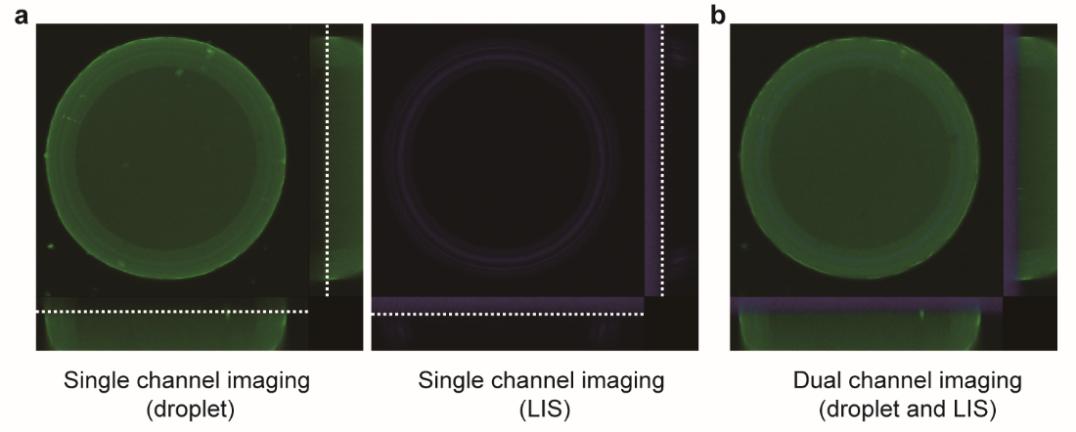


**Figure S5** 3D confocal images of water droplet on the LIS. a) Single channel imaging of droplet (Green) and LIS (Purple). The white dotted line represents the scanned cross-section of the droplet. b) Dual channel imaging of droplet on LIS.


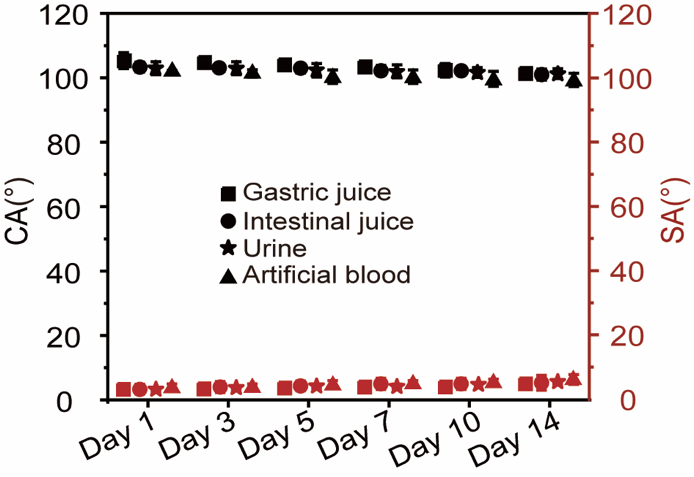


**Figure S6** Contact angle and sliding angle of LIS after immersion in various physiological fluids.


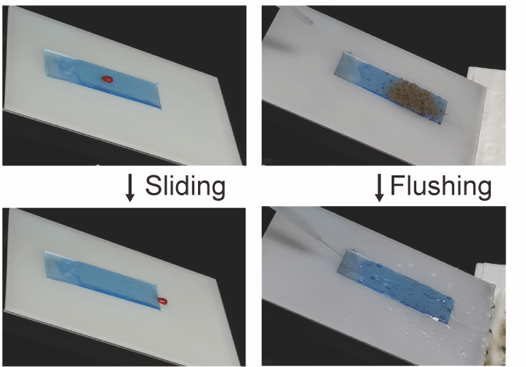


**Figure S7** Showing the antifouling performance of LIS, including blood repellency and wet dirt clean.


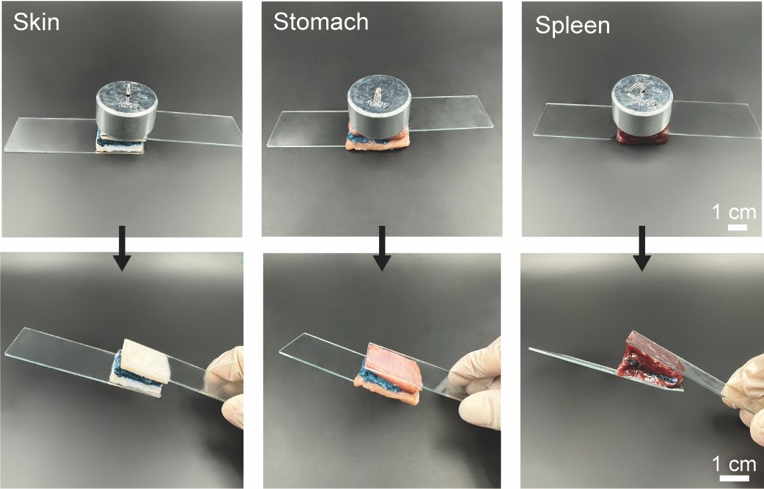


**Figure S8** Adhesion formation of powder-formed hydrogel to fresh tissues after 2 h contact.


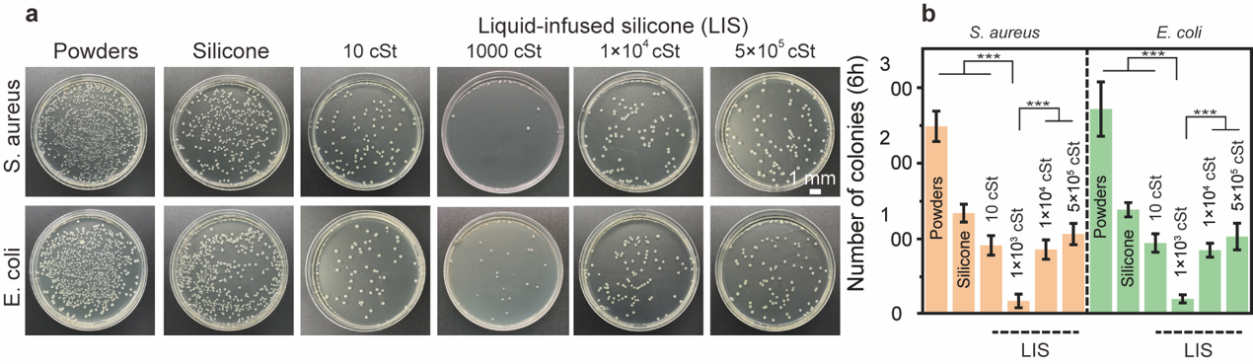


**Figure S9** Antibacterial attachment properties of powders, silicone, and LISs (incubated with the bacterial solution for 6 hours). a) Optical images of antibacterial adhesion performance of hydrated powders, silicone and LISs. b) Colony number of microorganisms formed on a solid medium.


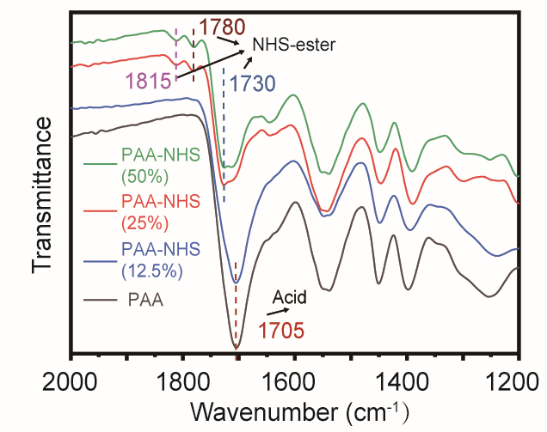


**Figure S10** Fourier-transform infrared spectroscopy (FTIR) of PAA and NHS grafted PAA.


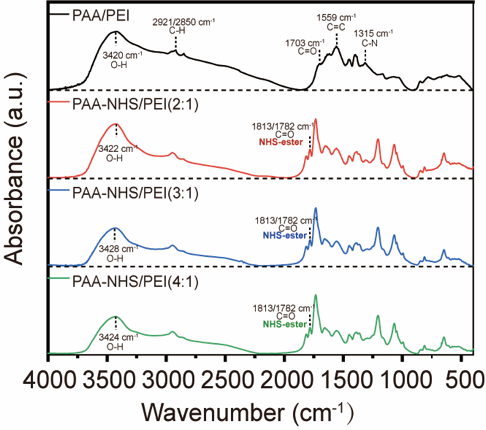


**Figure S11** Infrared spectral data of PAA-NHS/PEI and PAA/PEI powders.


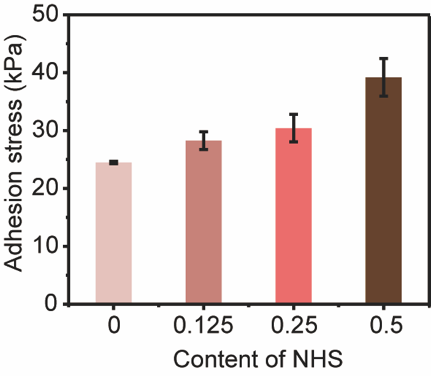


**Figure S12** Adhesion strength of hydrated powders with different content of NHS.


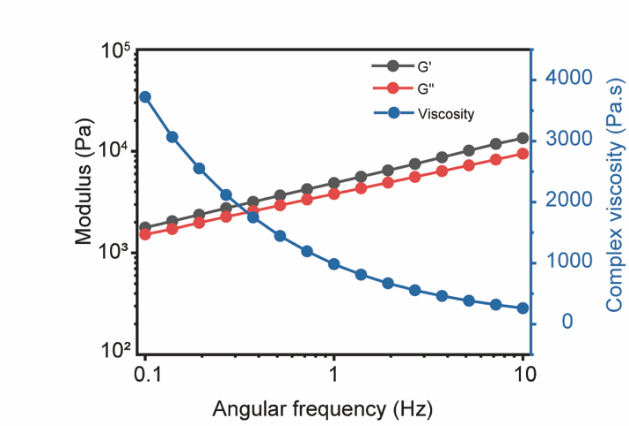


**Figure S13** Frequency sweep test of the PAA-NHS/PEI hydrogel.


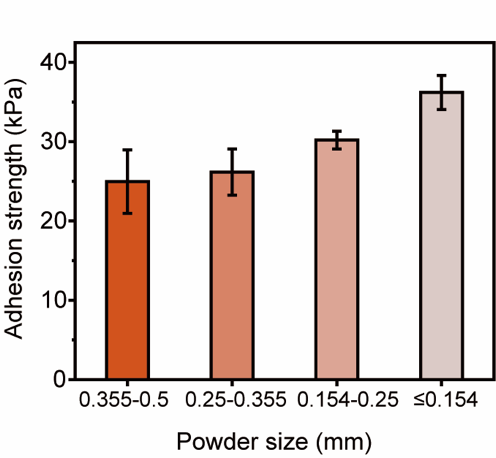


**Figure S14** Effect of PAA-NHS/PEI size on its adhesion strength.


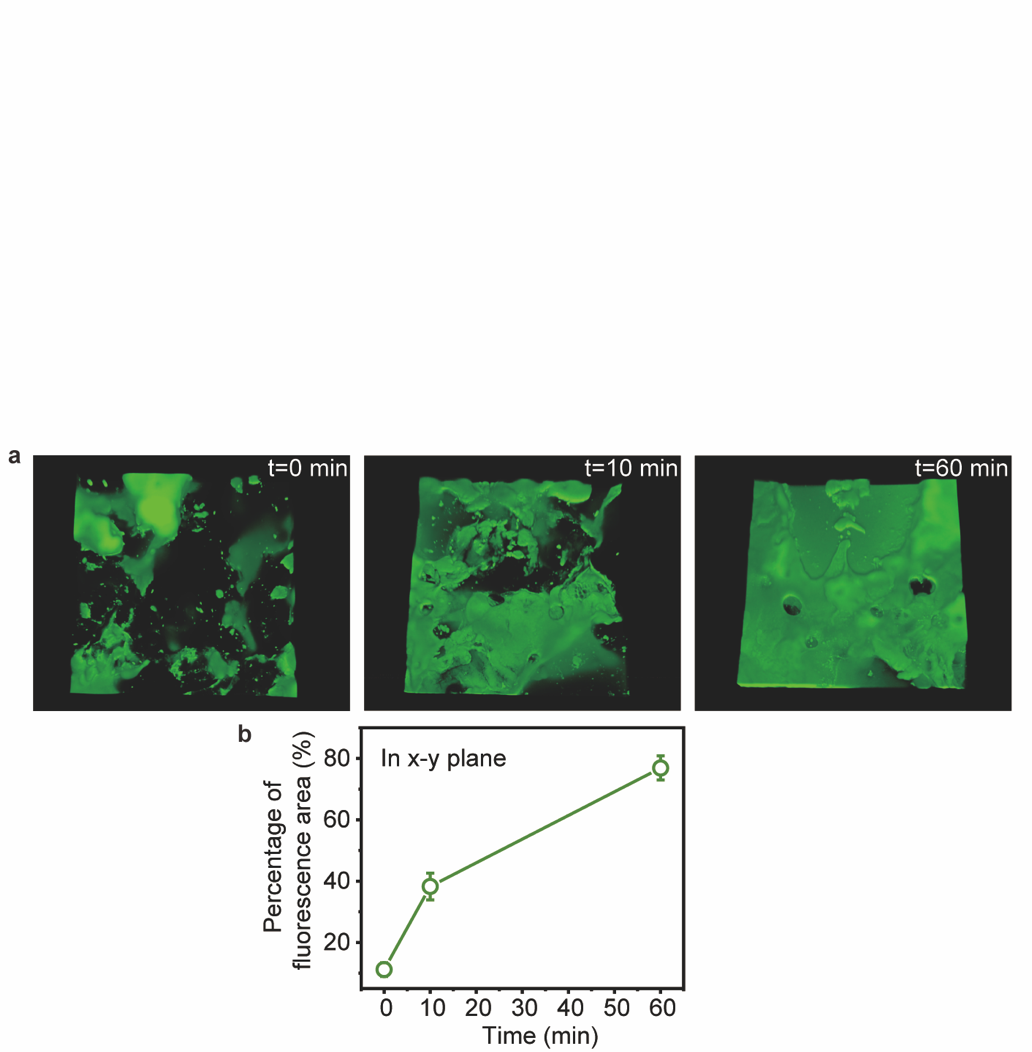


**Figure S15** Confocal images of hydrated PAA-NHS/PEI powders show whole hydrogel network is gradually formed because of polymer diffusion.


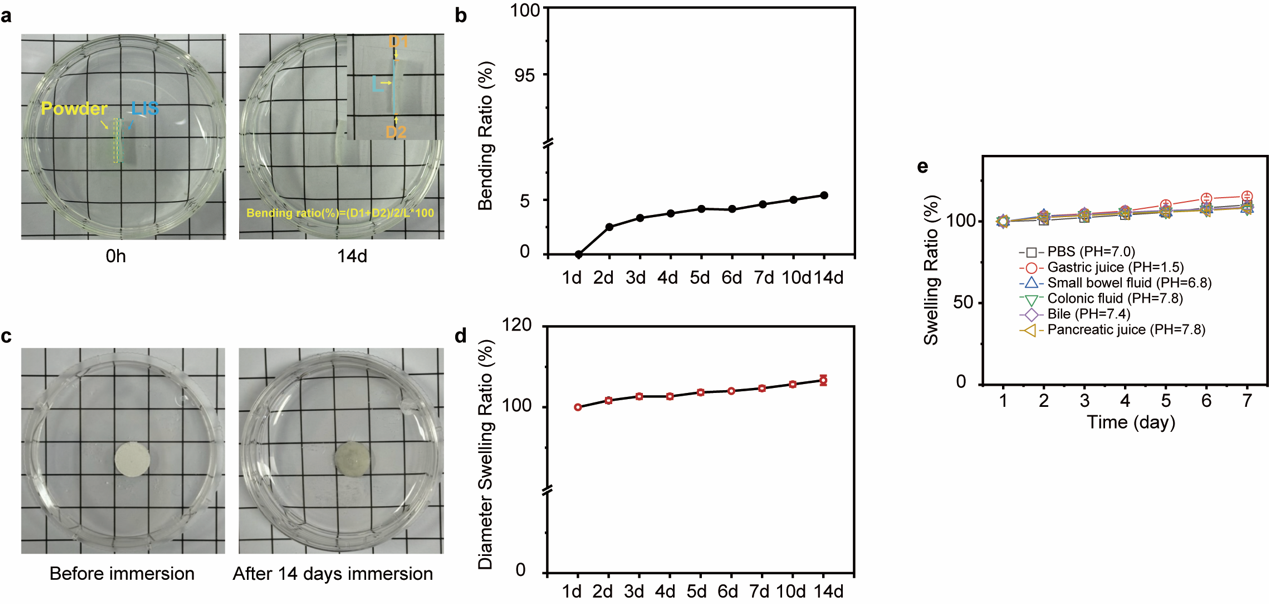


**Figure** **S16** a) Photographs of SAJG immersed in PBS buffer. b) Bending ratio of SAJG in PBS buffer. c) Photographs of PAA-NHS/PEI tablets before and after immersing in PBS buffer. d) Diameter swelling ratio of PAA-NHS/PEI tables in PBS buffer. e) Swelling ratio of adhesive powders in diverse medium with different pH.


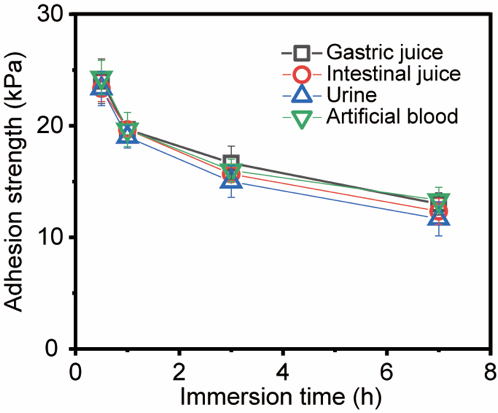


**Figure S17** In vitro adhesion strength of PAA-NHS/PEI powders after immersing in fluid environments.


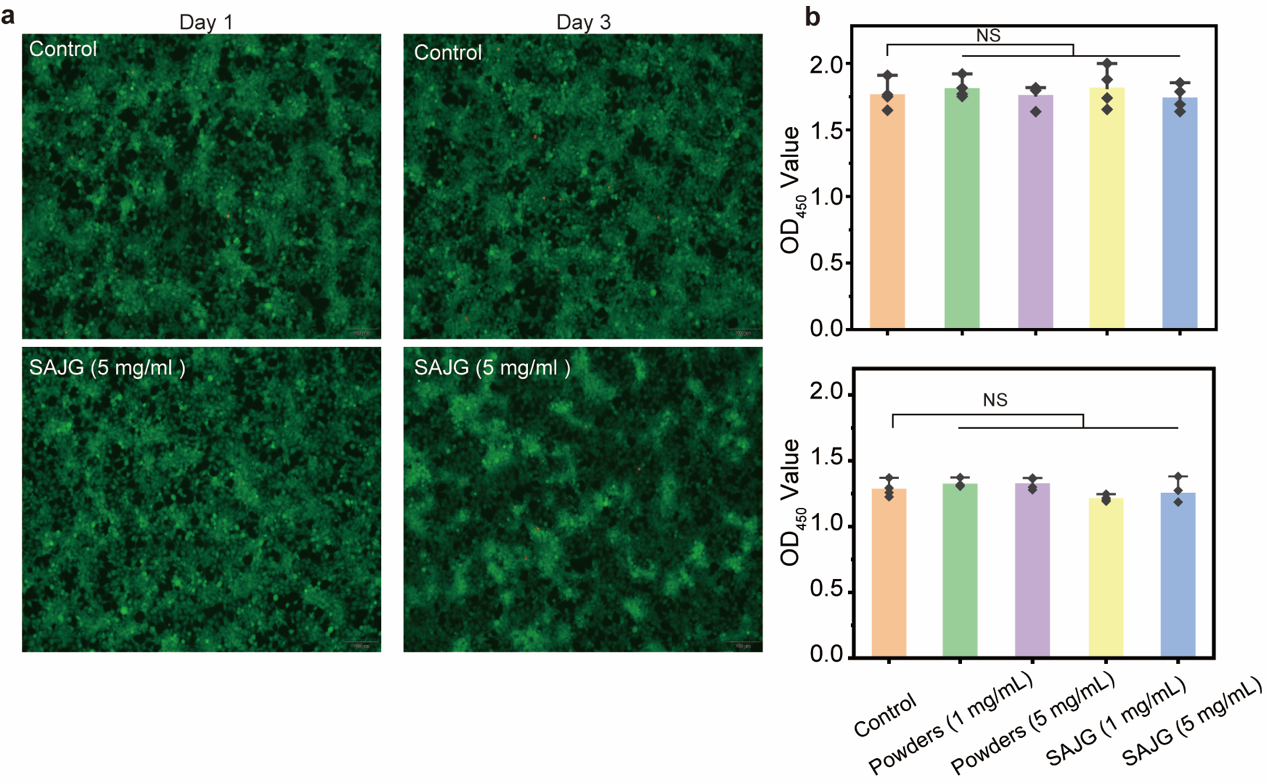


**Figure S18** Biocompatibility test of SAJG. a) Respective confocal images of stained live/dead L929 cells in the control group and in high concentration leachate of SAJG (5 mg/mL) after culture. b) Cell proliferation in control group and in different concentration of leachate after 1 and 3 days of culture (CCK 8).


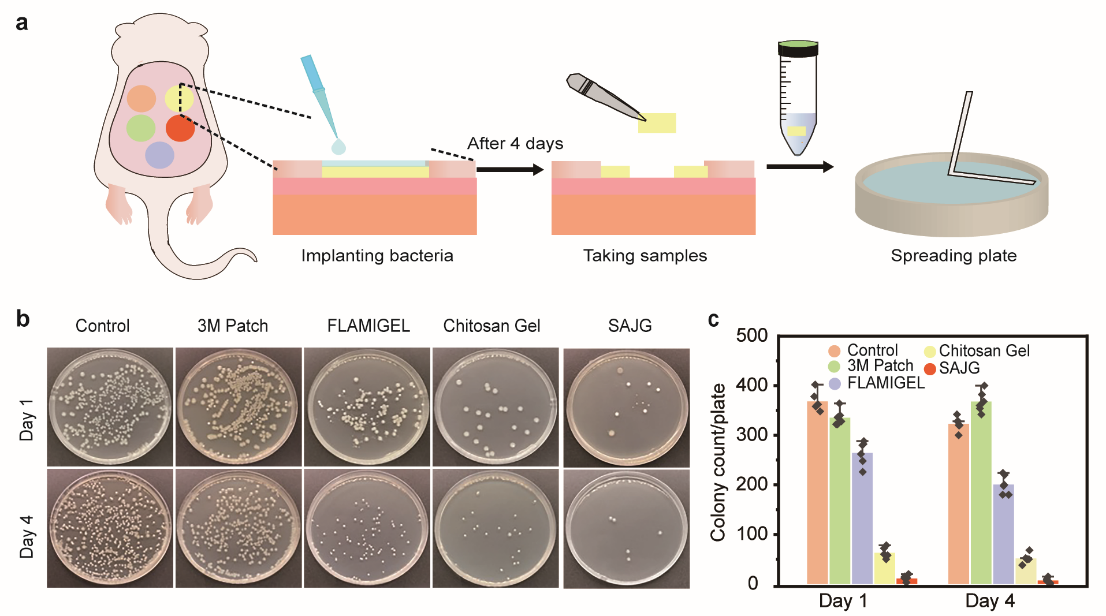


**Figure S19** Antibacterial ability of SAJG. a) Schematic illustration of bacterial infection model of rats and testing procedure of antibacterial ability. b) Optical images of bacterial colony of different samples. c) Activity of sample surfaces against S. aureus evaluated by counting bacterial colonies on agar plates.

**
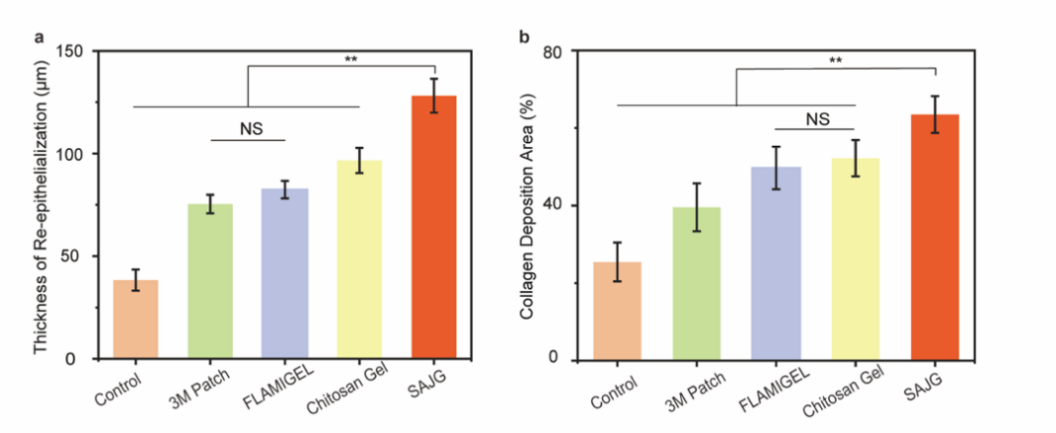
**

**Figure S20** a) Thickness of re-epithelialization and b) collagen deposition of wound on day 14.


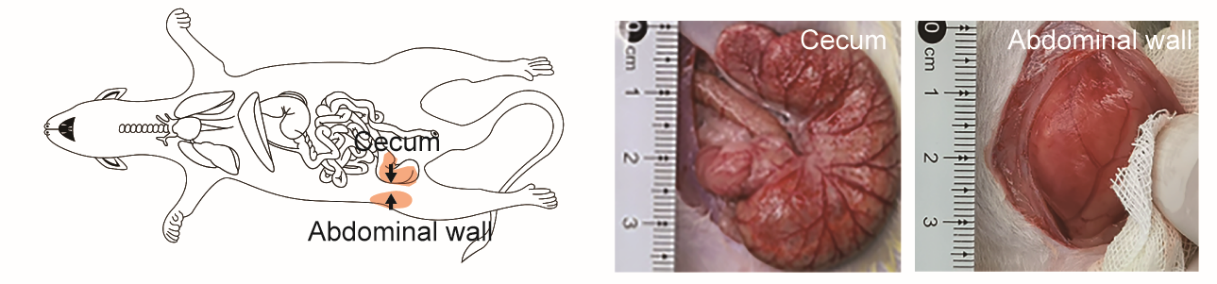


**Figure S21** The site and state of intestine damage, which was created by gauze rubbing.


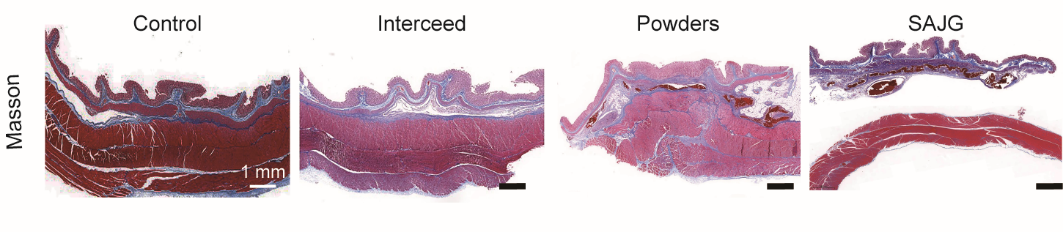


**Figure S22** Typical Masson’s trichrome staining images of tissue sections in different groups.
